# Assessment of clinical and therapeutic factors in patients with nasopharyngeal undifferentiated carcinoma

**DOI:** 10.1016/S1808-8694(15)30604-2

**Published:** 2015-10-18

**Authors:** Helma Maria Chedid, Sergio Altino Franzi, Rogério Aparecido Dedivitis

**Affiliations:** 1 Specialist in Head & Neck Surgery, Master's degree student in Health Sciences, Heliopolis Hospital - Hosphel, Sao Paulo. Surgeon of the Head & Neck Surgery and Otorhinolaryngology Department, Heliopolis Hospital - Hosphel, São Paulo.; 2 Doctor in Medicine, graduate course in Oncology, Medical School, Sao Paulo University. Faculty member in the graduation course Health Sciences, Heliopolis Hospital - HOSPHEL, So Paulo.; 3 Doctor in Medicine, graduate course in Otorrinolaringologia and Head & Neck Surgery, UNIFESP - Paulista School of Medicine. Faculty member of the graduation course in Health Sciences, Heliopolis Hospital - HOSPHEL, Sao Paulo. Full Professor, Otorrinolaringologia and Head & Neck Surgery, Santos Metropolitan University. Full Professor, Head & Neck Surgery Department, Fundacao Lusiada, Santos. Graduation Course in Health Sciences, Heliopolis Hospital - HOSPHEL, São Paulo.

**Keywords:** nasopharynx, carcinoma, chemotherapy, radiotherapy

## Abstract

The nasopharyngeal carcinoma (NPC) is a rare cancer with a high incidence in Southern Asia.

**Aim:**

to study the demographic, clinical, therapeutic, and prognostic factors of nasopharyngeal undifferentiated carcinoma in a reference service.

**Materials and methods:**

A retrospective study was made of 46 patients from January 1998 to August 2000. The patients had no previous treatment and did not present any evidence of synchronous tumors or distance metastases.

**Results:**

The age ranged from 14 to 78 years (mean = 46 years); 35 (76%) patients were male. All patients were Caucasian or African-Brazilian. The onset of initial symptoms ranged from 1 to 48 months (mean = 7 months); 47% of the subjects smoked tobacco and 33% consumed alcoholic beverages. A lump in the neck was the most frequent symptom (34 patients). Twenty-two patients were clinically staged as T1/T2 and 24 patients as T3/T4; 24 patients were classified as N2, and 16 patients were staged as N3. Curative treatment consisted of radiotherapy and simultaneous chemotherapy in clinical stages III and IV. Of 27 patients that were monitored, 52% were alive with no evidence of disease after three years.

**Conclusion:**

All patients were in advanced clinical stages of the disease. The three-year disease-free survival rate was 52%.

## INTRODUCTION

The incidence of nasopharyngeal carcinomas is rare in most of the world, except among Southeastern Asian populations, where it is one of the most frequent malignancies. The highest incidences are seen in Alaskan and Greenland Eskimos and in Tunisia. The incidence remains high in Asians who have migrated to Western countries, compared to Caucasians.^1^, ^2^ The etiology is still unknown. There are a few hypotheses, such as hereditary causes related to the HLA-A2 and HLA-Bsin^2^ viral genotypes, Epstein-Barr virus (EBV) infection, altered chromosomes and cultural factors such as increased amounts of fish and preserved food consumption that are rich in nitrosamides, known environmental carcinogens.^3^, ^4^ The EBV may be found in tumor cells of patients by an RNA signal,5 suggesting that peripheral blood may be investigated in endemic areas of this disease to detect anti-EBV antibodies. As opposed to other head and neck malignancies, smoking and alcohol consumption appear to be less important in the carcinogenesis of the aforementioned tumors. The prognosis of nasopharyngeal tumors depends on initial clinical staging and the histological subtypes.^3^

The incidence is distributed across all age groups, predominating at 50 and 60 years; males are affected more often. The incidence reaches 10 to 20 per 100 thousand male inhabitants and 5 to 10 per 100 thousand females in certain Southeastern regions of China.^6^

The diagnosis tends to be made when the disease is advanced, including signs and symptoms of invasion of the cranial base and cranial nerve involvement.^7^ Symptoms in early disease are rare, and may include nasal block, headaches, deafness and otitis media. In later phases of the disease, invasion of adjacent structures to the cranial base affect more often the maxillary branch of the trigeminal nerve and the abducens nerve.^7^

The incidence of neck metastases upon the initial presentation is high, and may be the first sign of the disease in 44% to 57% of cases. Bilaterally involved neck lymph nodes are found in 50% of cases of undifferentiated carcinomas.^8^ A study of over 5,000 nasopharyngeal carcinoma patients showed that 29% had distance metastases and 17% of these presented lesions exclusively in distant sites.^9^

The initial treatment of choice is radiotherapy for tumors at early stages, and radiotherapy with chemotherapy for tumors at advanced stages.^10^, ^11^ Salvage surgery is done for the removal of large neck metastases. Salvage surgery in persistent primary tumors or recurring tumors is rarely indicated.

The purpose of this study was to assess the oncological results of treating nasopharyngeal carcinomas during a 20-year period in a reference head and neck unit.

## SERIES AND METHOD

This was a retrospective study that surveyed the medical files of 46 patients diagnosed histopathologically with undifferentiated nasopharyngeal carcinomas between January 1978 and August 2000. The Research Ethics Committee of the institution approved the study (number 550).

Inclusion criteria were patients with untreated disease, a confirmed histopathological diagnosis, absence of other synchronic neoplasms, absence of distance metastases upon the initial presentation, and initial therapy with curative intent. Between 1978 and 1985 undifferentiated carcinomas were diagnosed by histopathology, because immunohistochemical testing only became available at the institution from 1985 onwards.

The diagnosis of disease was made based on locoregional examination and exams such as nasofibrolaryngoscopy and computed tomography of the face. The definite diagnosis of all cases of undifferentiated nasopharyngeal carcinomas was made by the pathological examination (HE staining) and immunohistochemistry of biopsies of nasopharyngeal lesions, taken by posterior rhinoscopy or nasosinusal endoscopy.

Teletherapy (cobalt unit and linear accelerator) was used for radiotherapy. The radiation dose ranged from 65 to 75Gy on the primary tumor, divided into 1.8 Gy/day, five days a week. Neck lymph node chains received at least 45 Gy to 50 Gy.

Simultaneous chemotherapy was done with cisplatin (CDDP), 100 mg/m2/week, infused intravenously in one hour. Chemotherapy was given by the arterial route in one case.

The following variables were assessed: age of onset of disease, race, clinical presentation at the time of the diagnosis, duration of the clinical history between the first symptom or sign and the first visit to a specialist, the initial therapy, local and regional recurrences, and distance metastases.

## RESULTS

The age of patients treated for undifferentiated nasopharyngeal carcinomas ranged from 14 to 78 years (mean 46 years). There were 35 male patients (76%) and 11 female patients (23%). All patients were either Caucasian or mixed color/black. There were no patients of Asian origin.

The mean time elapsed between the first symptoms and the first visit to a specialist was seven months (1 to 48 months). There were 47% smokers and 33% users of alcoholic beverages.

The most commonly reported symptoms were the presence of a lump in the throat (34 patients), followed by nasal bleeding (10 patients), nose block (seven patients), and pain and weight loss (three patients).

Clinical staging was based on the International Tumor Classification (AJC-UICC); all 46 patients were restaged according to the latest revision done in 2002. Relative to the primary tumor, 10 cases were T1; 12 cases were T2; eight cases were T3, and 16 cases were T4. Relative to neck metastatic lymph nodes, five patients were N0, seven patients were N1, nine patients were N2, and 25 patients were N3. Clinical staging (CS) showed that no patient was EC I or II, 16 patients were EC III, and 30 patients were EC IV. The locoregional exam showed that 13 patients (28.2%) presented lesions on the nasopharyngeal roof. In 24 patients (52.1%), lesions were on the lateral walls. In nine patients (19.5%), the primary lesion was not located in the medical examination, being diagnosed by image exams.

The physical examination suggested an advanced primary tumor with cranial base or intracranial invasion in seven patients (15.2%). Paralysis of the 6th cranial nerve was seen in three patients (6.5%); paralysis of the 9th cranial nerve was seen in two patients (4.3%); and paralysis of the 10th cranial nerve was seen in two patients (4.3%). There was no paralysis in more than one cranial never in these seven patients.

Therapy in all patients was conventional external radiotherapy. Concomitant chemotherapy was done in six patients (13%). Chemotherapy was given by the intra-arterial route in one patient (16.6%); the remaining five patients (83.4%) received systemic intravenous chemotherapy.

The radiotherapy dose on the primary tumor ranged from 60 to 70 Gy; the radiotherapy dose on neck lymph node chains ranged from 34 to 50 Gy. An extra dose (boost) was done postoperatively on the dissected surgical bed in three of four patients that underwent neck salvage surgery.

Follow-up was done for 27 (58.7%) of 46 patients. Of these 27 patients, four (14.8%) underwent neck salvage surgery due to persistent or recurrent palpable neck lymph nodes.

Distance metastases were diagnosed in seven patients (25.9%) during a 296 month follow-up period. There was locoregional control of the disease and involvement of the brain, bones, lungs and skin.

**Figure 1:**
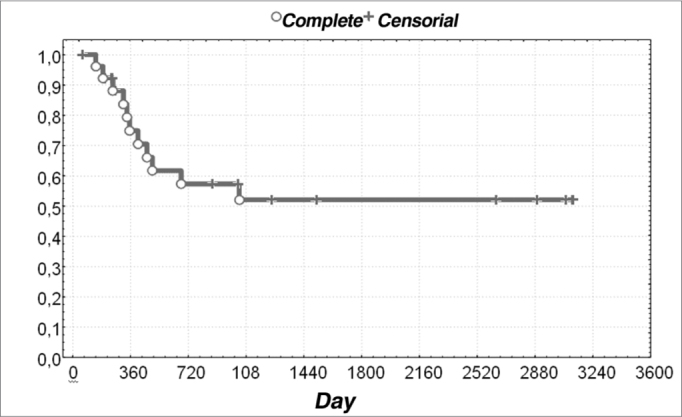
Distribution of disease-free survival of patients with undifferentiated nasopharyngeal carcinomas.

Of the 27 patients that were followed-up after the initial treatment, 52% were alive and disease-free at three years. The disease-free survival rate was 52% (Figure 1).

## DISCUSSION

According to the World Health Organization, nasopharyngeal carcinomas may be divided into three types, as follows: type 1 - epidermoid carcinomas, which occurs mostly in adults; type 2 - non-keratinized carcinomas; and type 3 - undifferentiated carcinomas. Type 1 occurs in less than 5% of cases in endemic areas, and in 25% of patients in non-endemic areas. Type 1 is also the least sensitive to radiotherapy; its prognosis is the worst.^8^

In our study most of the patients were in the fifth decade of life. There was no bimodal distribution (a first peak in adolescents and young adults). Race was a second variation compared to the literature; most of the patients were Caucasian, and there were no patients of Asian origin.^12^, ^13^ These findings coincide with those of the National Cancer Institute (USA), showing a higher incidence of nasopharyngeal carcinomas in young adult Caucasians and Afro-Americans compared to Asian patients.^14^

Tobacco and alcohol are relevant etiological factors in upper digestive airways. Undifferentiated nasopharyngeal malignancies, however, are less affected by these substances, which are not considered etiological in these cases.^15^

Radiotherapy and chemotherapy are the preferred therapies in nasopharyngeal carcinomas. Radiotherapy is the main treatment in initial tumors. Chemotherapy is added in cases with advanced tumors.^10^, ^16^

Computed tomography and magnetic resonance imaging currently have a significant role when planning radiotherapy for nasopharyngeal tumors. A study of 275 magnetic resonance exams showed that there was extension of metastases to the retropharynx in 63.6% of cases,^17^ which is relevant when planning therapy.

The side effects of radiotherapy have decreased with the advent of other forms of irradiation (brachytherapy and stereotaxic radiotherapy) compared to conventional external radiotherapy. The most frequent side effects are xerostomy, otitis media, trismus and severe neck fibrosis.^18^

A randomized study of 229 patients with advanced stage tumors showed that radiotherapy alone or with chemotherapy did not improve local and regional control indices.^19^ Another randomized trial from the Intergroup Study 0099 demonstrated improved disease remission rates by using chemotherapy with radiotherapy in advanced stage tumors; in this study, 3-year disease-free survival was 69% compared to 24% when treated with radiotherapy alone.^10^

The Asian Oceanian Clinical Oncology Association found no benefits in using neoadjuvante chemotherapy with radiotherapy in advanced nasopharyngeal carcinomas. They did find a lower incidence of local and regional recurrences, but no change in the disease-free survival.^20^

The literature on control of this disease presents mixed results. The first published studies had control rates of 87% in T1, 94% in T2, 68% in T3, and 44% in T4 cases relative to the primary tumor.^21^ Later papers showed improved control rates for advanced tumors, with 73% and 100% in T3, and 71% and 63% in T4.^22^, ^23^ For the N stage, disease control rates are similar in a number of studies, reaching 92% and 90% for N0, 87% and 88% for N1, and 89% and 82% for N3.^22^, ^23^

The incidence of local recurrence ranges from 18% to 58%. Therapy consists of reirradiation or salvage surgery, the choice of which remains controversial. Reirradiation implies in more complications such as cerebral necrosis, hypothalamic-pituitary failure, deafness, retinopathy, transverse actinic myelitis and cranial nerve dysfunction. There have been fewer complications by using brachytherapy and stereotaxic radiotherapy. Control of local recurrence with a second round of radiotherapy has been reported in 10% to 30% of cases.^24^

Regional recurrence rates vary from 8% to 34% of cases, similar to the cases of lymph node disease persistence at the end of therapy. In these cases, salvage neck dissection is the main form of treatment; success rates reach 66% of cases. Even with regional disease control, patients tend to course with distance metastases.^8^, ^23^

Neck dissection following the initial therapy used to be recommended in all cases of pre-radiotherapy extensive lymph node disease.25 In a series with 27 patients that underwent programmed neck dissection, all cases had positive lymph nodes in pathology; 84% of cases had extracapsular rupture.

The incidence of hematogenic metastases is high; they commonly occur following locoregional disease control. Metastases at a distance arise in 5% to 10% of patients upon the diagnosis. Frequent sites include the lungs, bones and liver.26 A study of 900 patients demonstrated metastases at a distance in 22.2% of cases during the follow-up period of patients that had local and regional disease control.^27^

The treatment of choice in our unit is radiotherapy alone in stages I and II, and radiotherapy with chemotherapy in stages III and IV. In this paper only six patients were also treated with chemotherapy in advanced disease. During the timeframe of this study, radiochemotherapy for nasopharyngeal carcinomas in clinical stages III and IV was recommended as the ideal treatment; this measure eventually became the therapeutic guideline. In cases with large unilateral or bilateral neck metastases, in which planning radiotherapy is difficult, neoadjuvante chemotherapy is used from 1 to 3 cycles to reduce the size of such metastases, after which radiochemotherapy is done. The indication of neoadjuvante chemotherapy is based on some papers in the literature that have demonstrated its benefits; in such cases, toxicity is low and there is an increase in disease-free survival.^28^, ^29^

Neck surgery is done if lymph nodes persist, if there are large metastases with probable extracapsular extravasation that responded to therapy with complete clinical remission, in salvage of cervical lymph nodes (especially in N3 cases) and in salvage neck dissection following recurrences.

## CONCLUSION

The distribution of undifferentiated carcinoma did not follow a bimodal distribution per age group. All of the treated patients were in advanced clinical stages (III and IV), and had a 52% disease-free survival rate at three years.
